# Abdominal aortic aneurysm and acute appendicitis: a case report and review of the literature

**DOI:** 10.1186/s13256-021-02703-x

**Published:** 2021-04-17

**Authors:** Rubén Peña, Sergio Valverde, José A. Alcázar, Paloma Cebrián, José Ramón González-Porras, Francisco S. Lozano

**Affiliations:** 1 Servicio de Angiología y Cirugía Vascular, Complejo Asistencial Universitario de Salamanca (CAUSA), Instituto de Investigación Biomédica de Salamanca (IBSAL), Universidad de Salamanca (USAL), Paseo de San Vicente 139, 37007 Salamanca, Spain; 2 Servicio de Cirugía General y del Aparato Digestivo, Complejo Asistencial Universitario de Salamanca (CAUSA), Instituto de Investigación Biomédica de Salamanca (IBSAL), Universidad de Salamanca (USAL), Salamanca, Spain; 3 Servicio de Radiodiagnóstico, Complejo Asistencial Universitario de Salamanca (CAUSA), Instituto de Investigación Biomédica de Salamanca (IBSAL), Universidad de Salamanca (USAL), Salamanca, Spain; 4 Servicio de Hematología, Complejo Asistencial Universitario de Salamanca (CAUSA), Instituto de Investigación Biomédica de Salamanca (IBSAL), Universidad de Salamanca (USAL), Salamanca, Spain

**Keywords:** Abdominal aortic aneurysm, Appendicitis, Elderly patients, Synchronous pathologies

## Abstract

**Background:**

Abdominal aortic aneurysm and acute appendicitis occur relatively frequently in elderly patients. However, the co-occurrence of the two pathologies is very rare and serious.

**Case presentation:**

We present the case of an elderly Caucasian patient who was aware of having an abdominal aortic aneurysm but refused treatment and was subsequently admitted to the hospital’s emergency department with acute abdominal symptoms. A computed tomography scan raised the possibility of complication due to the characteristics of the aneurysm. The patient then agreed to emergency surgery. Laparotomy revealed the existence of an acute perforated appendicitis with a significant abscess in the right iliac fossa and an uncomplicated aneurysm. Appendectomy was performed and the abscess drained. The postoperative period passed without complications, and the patient again refused surgery for the aneurysm, which due to its anatomical characteristics was not a candidate for standard endovascular treatment.

**Conclusions:**

In light of this experience, we review the literature about the relationship between abdominal aortic aneurysm and acute appendicitis.

## Background

Abdominal aortic aneurysm and acute appendicitis are relatively common pathologies in elderly patients [1, 2]. However, the association of the two entities is very rare and serious [3].

Following our treatment of a patient with this association, in addition to presenting and discussing the clinical case, we review the literature on this unusual and complex relationship between abdominal aortic aneurysms and acute appendicitis.

## Case presentation

The case report concerns an 85-year-old Caucasian patient with a personal history of arterial hypertension, but no other medical background of interest. In 2015, the internal medicine service of her local hospital requested computed tomography (CT) scans of the thorax-abdomen-pelvis with and without intravenous contrast (26 January). The most significant findings of this exploration were: (1) a pulmonary granulomatous condensation, (2) an uncomplicated cholelithiasis, and (3) an abdominal aortic aneurysm (AAA), which started 3 cm below the renal arteries, with dimensions of 6.2 cm (antero-posterior) × 6.3 cm (transversal) × 10.0 cm (cranio-caudal), and a 2.4-cm-thick mural thrombus. The patient refused surgical treatment for the aforementioned aneurysm.

On 18 December 2018, the patient was referred from her regional hospital to the emergency department of our hospital because of the presentation of abdominal pain, with a diagnosis of complicated AAA. On arrival (3.00 a.m.), the patient was conscious and some vital signs (blood pressure, heart rate, respiratory rate, and temperature) were within the normal range. At that time, she reported that a few hours before she had experienced sudden onset of abdominal pain, located in the epigastrium and radiating to both iliac fossae and located in the left iliac fossa. The most pertinent analytical results included a discrete leukocytosis (11.03 × 10/uL) with neutrophilia (88.1%), elevated C-reactive protein (6.45 mg/dL), and a normal level of procalcitonin (0.17 ng/mL). Otherwise, the patient had normal blood count, coagulation, biochemistry (including troponins), and blood gases.

**Fig. 1:**
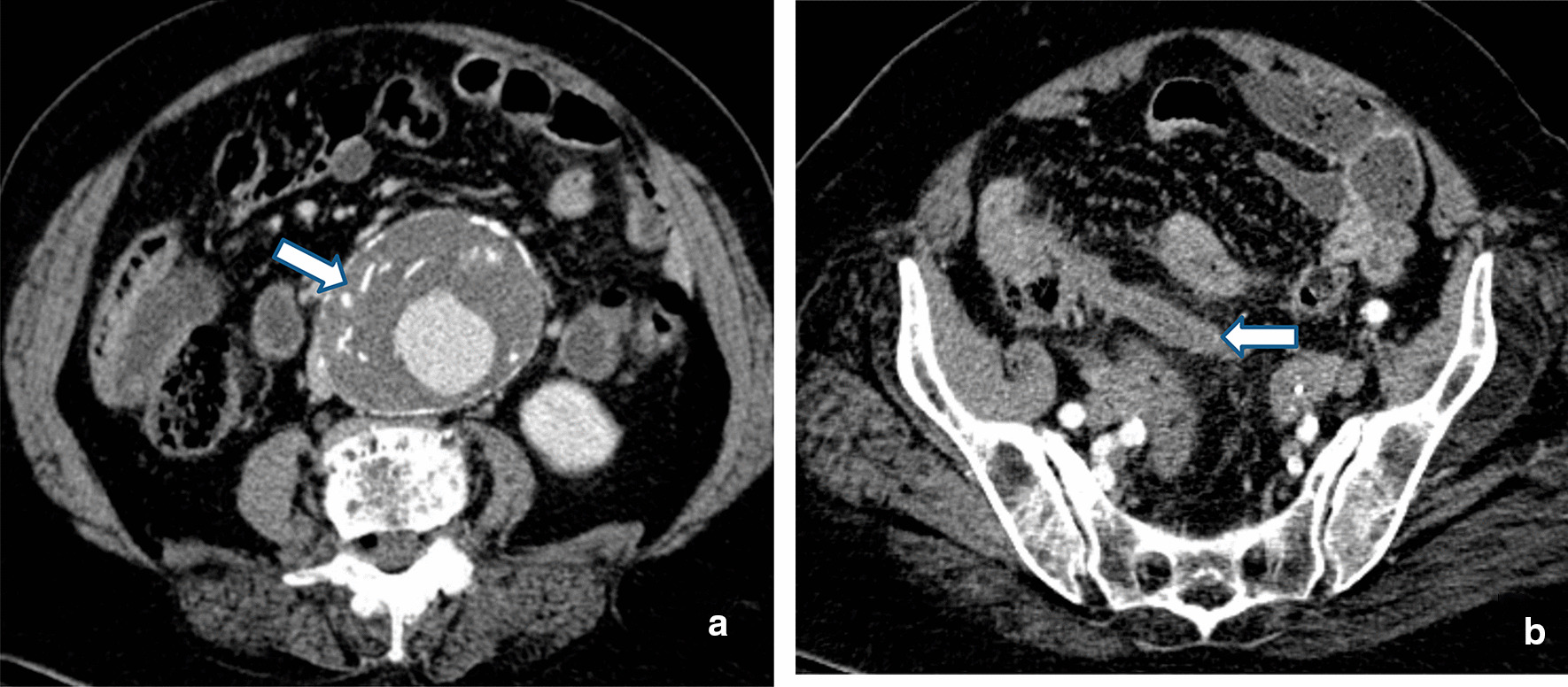
Preoperative computed tomography (transverse sections): **a** Aortic abdominal aneurysm of 8 cm (antero-posterior) × 8.5 cm (transversal), without signs of rupture (no free liquid or signs of retroperitoneal hematoma are visible), and radiological signs of intrathrombal hemorrhage (arrow). **b** Thickening of the sigmoid wall and slight increase in the echogenicity of the adjacent fat (5 cm in length). Inflamed appendix and increased echogenicity of adjacent fat (arrows)

An angio-CT was performed, which showed the known AAA to have measurements of 8 cm (antero-posterior) × 8.5 cm (transversal) × 10 cm (cranio-caudal), without signs of rupture (no free fluid or signs of retroperitoneal hematoma were observed), but with radiological signs of intraluminal thrombus hemorrhage (Fig. 1a). A 2.2-cm transverse growth of the aneurysm since the CT performed 3 years before (at the time of diagnosis) was also observed. Jointly, in the pelvis, a 5-cm segment of the sigmoid colon presenting a thickening of the wall was observed. A slight increase in the echogenicity of the adjacent fat suggested the action of an inflammatory process (diverticulitis or non-specific colitis).

**Fig. 2:**
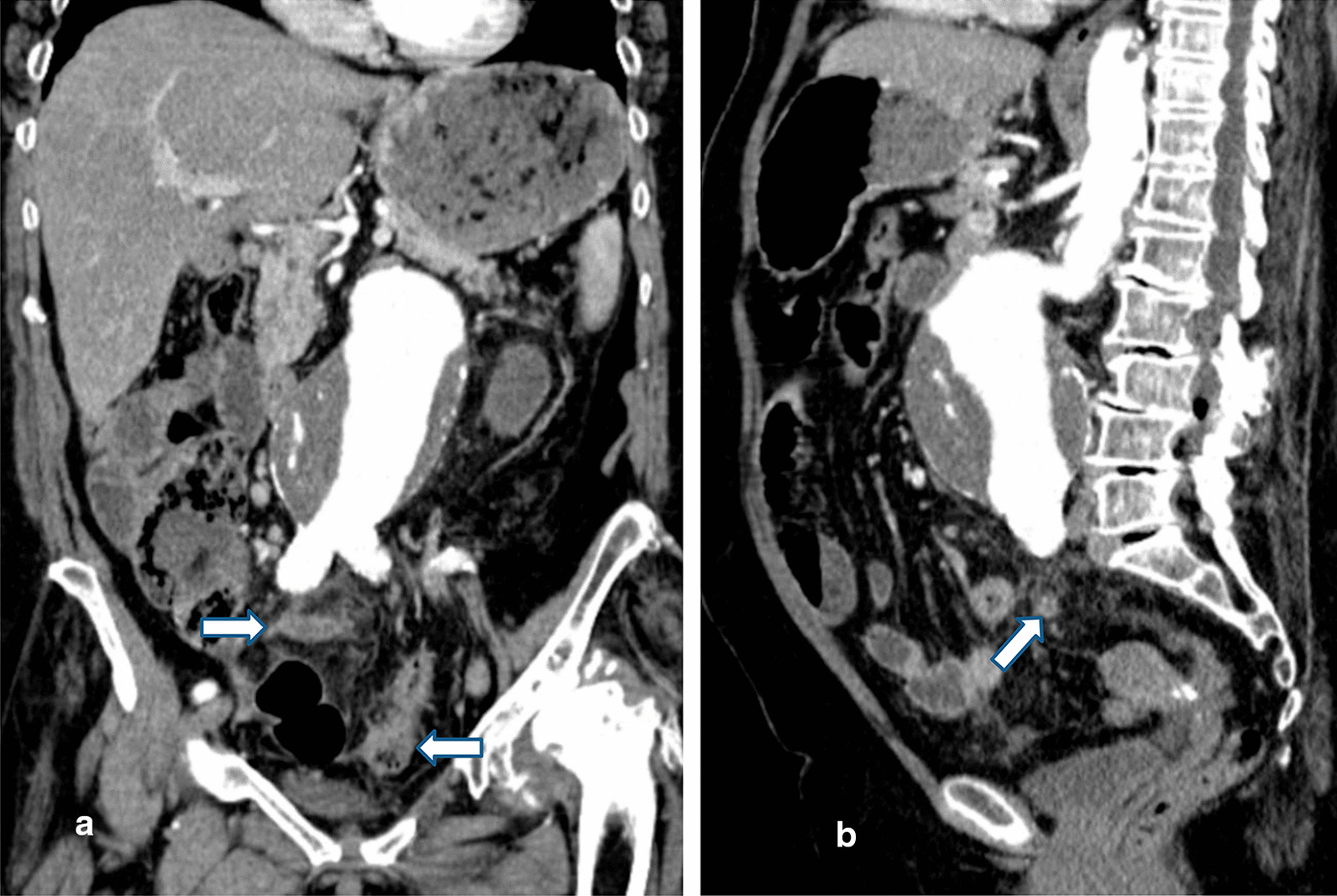
Preoperative computed tomography (coronal and sagittal sections): **a** inflamed appendix and increased echogenicity of adjacent fat (arrows). **b** Thickening of the sigmoid wall and slight increase in the echogenicity of the adjacent fat (arrow)

An uncomplicated cholelithiasis (already known from the previous CT) was noted. Finally, our center’s radiologist noted an inflammation of the appendix and an increase in the echogenicity of the adjacent fat (Figs. 1b and 2a, b).

During the observation period, the patient was stable, although she had a fever with a peak temperature of 38°C. A second analysis showed an increase in leukocytosis (13.23 × 10/uL) with almost identical neutrophilia (87.2%), hyperglycemia (179 mg/dL), alteration of the ionogram (hyponatremia of 134 mmol/L, hypokalemia of 2.9 mmol/L, and hypochloremia of 95 mmol/L), elevated reactive-C protein (17.55 mg/dL), and procalcitonin above the reference values (0.53 ng/mL). All other analytical results were unchanged.

At this time, the patient was evaluated by the general surgery and gastroenterology services, which concluded that the clinical results and examination were very nonspecific. Therefore, the available data pointed most strongly toward a symptomatic and unstable AAA with a high risk of imminent rupture. Emergency surgery was therefore proposed to treat her AAA, to which the patient gave her consent. The anatomical characteristics of the neck of the aneurysm (short and with severe angulation) compelled us to rule out standard endovascular surgery.

Fourteen hours after admission, the vascular surgery team performed a xyphopubic laparotomy. There was a gangrenous appendix (with microperforations) and purulent free fluid in the right iliac fossa. The general surgery team on duty was notified that they should perform an appendectomy, wash out the purulent fluid, and take samples for culture. Given the high probability of infection of the prosthesis (to treat AAA) and the absence of retroperitoneal hematoma suggestive of complication, it was decided not to treat the AAA during this surgical session.

Broad-spectrum antibiotic therapy was prescribed until the result of the culture had been received. This revealed the presence of a strain of *Proteus mirabilis* that was sensitive to amoxicillin-clavulanic acid, gentamicin, and ceftriaxone.

**Fig. 3:**
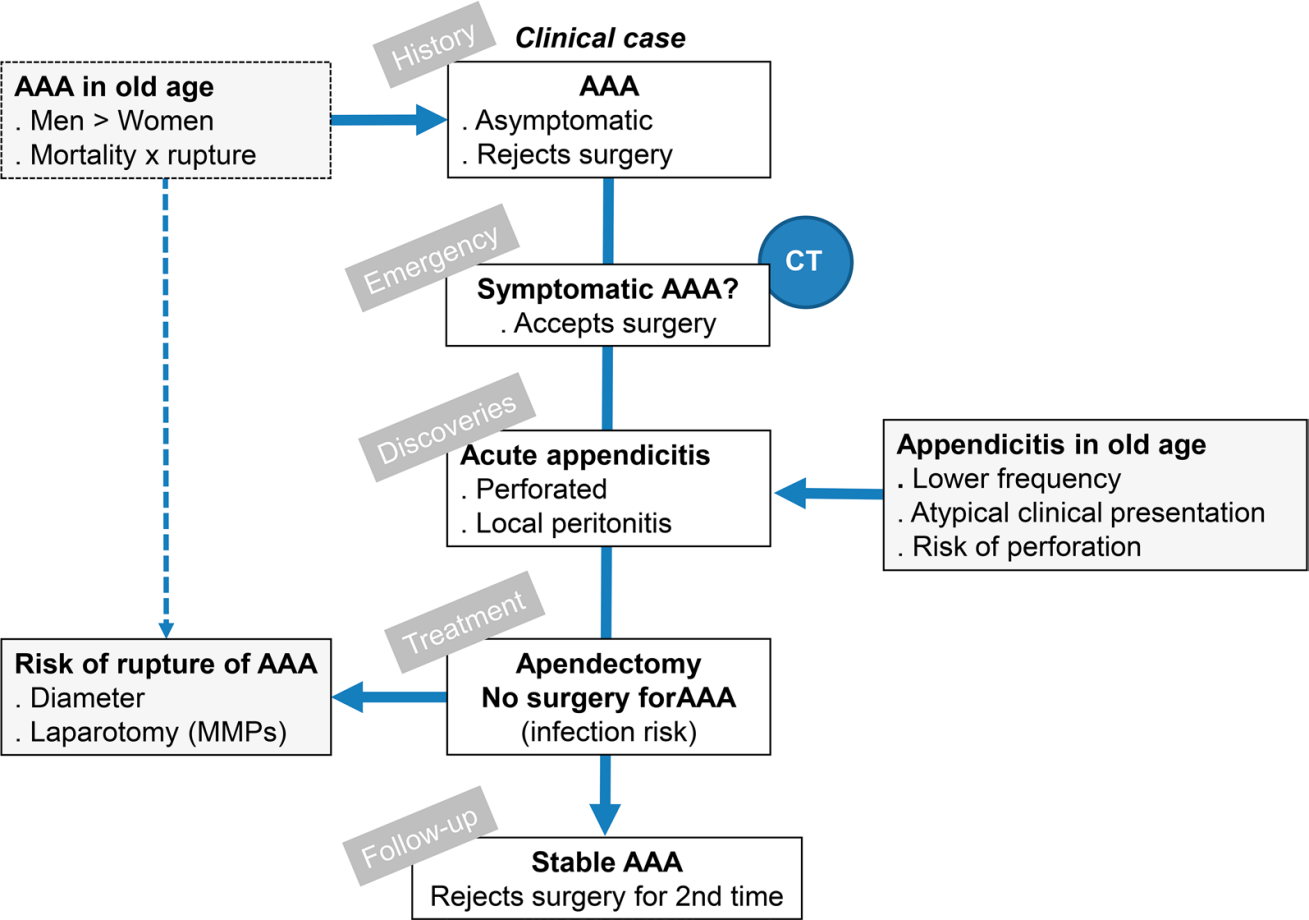
Diagram of the patient and her problem

The patient progressed favorably and was discharged a week later. She was re-evaluated 1 and 4 months after discharge (24 January and 12 April 2019) in the vascular surgery clinic. The patient again rejected scheduled surgical treatment of her aortic aneurysm (Fig. 3), after being informed in detail about the risk of AAA rupture and death. At the present time (31 August 2020), the patient, 87 years old, remains alive 56 months from the diagnosis of the aneurysm and 20 months after the referred appendectomy.

## Discussion

Abdominal aortic aneurysm and appendicitis are two of the many abdominal pathologies that may affect the elderly. These pathologies usually occur in isolation, but in rare cases they can be associated, where upon their severity increases.

### Abdominal aortic aneurysm (AAA)

AAA is a pathology occurring almost exclusively in the elderly population, and preferentially in males. Patients are initially asymptomatic, and when symptoms appear they are usually nonspecific (abdominal or back pain). Without treatment, its natural history leads to the growth and rupture of the aneurysm, which has a high mortality rate. In the case of breakage, the pain at the abdominal, back or flank level is more intense; the classic triad (hypotension, back pain, and palpable mass) is only present in 25–50% of cases [1], and the symptomatology is often more oriented towards cholecystitis, lumbago, or renal colic. For these reasons, in atypical cases, the diagnosis of ruptured/leaking AAA is sometimes incorrect or delayed [2]. Obviously, an ultrasound or a CT would clarify these doubts.

#### Indication for surgery

There is a broad consensus about the indication for elective AAA treatment (open or endovascular surgery). This is based on three criteria: (1) maximum transverse diameter of AAA (≥ 5.5 cm for men and ≥ 5.0 cm for women); (2) presence of symptoms; (3) AAA growth (> 1 cm/year) [4].

In our patient, there was a clear indication to operate (initial transverse diameter of AAA = 6.3 cm and 8.5 cm at the time of appendicectomy), but the patient refused treatment because she did not accept the risk of open surgery, which would have been necessary because the anatomical conditions of the AAA did not allow standard endovascular treatment.

#### AAA rupture factors

A review has summarized the existence of numerous biological (large diameter and annual growth of the aneurysm), clinical (female gender, high blood pressure, chronic obstructive pulmonary disease, smoking habit), and biomechanical and enzymatic (matrix metalloproteinases, MMPs) factors related to AAA rupture [5]. A recent systematic review has defined the relevance of different potential factors (circulatory, biomechanical, and genetic) related to the growth and rupture of an AAA. According to this review, two factors have been identified that increase the risk of rupture: stress on the wall and the diameter of the aneurysm [6].

Nine studies show that the diameter of the AAA is an important marker of rupture [6]. There is even agreement, based on data from several studies, about how the annual risk of rupture of an AAA is related to its diameter: for those measuring 40–49 cm, the annual risk is 1%, while if the diameter exceeds 7 cm, the figure rises to 30–33% [4].

The aortic wall contains certain structural proteins (collagen and elastin) and their activators and inhibitors (collagenase and elastase). Therefore, and with respect to the stress on the aortic wall, it has been shown that an increase in the level of certain matrix metalloproteinases (MMPs) or their inhibitors increases the risk of aneurysm rupture. Specifically, MMP-9 and the MMP-1 inhibitors are markers of rupture [7, 8].

In this context, in 1980, Swanson *et al.* [9] were among the first to draw attention to the fact that laparotomy is a precipitating factor for the rupture of abdominal aneurysms. The authors explain this in terms of a reduction in the collagen content of the wall of the aneurysm, which causes it to weaken. There is usually a balance between collagen synthesis and lysis, but the latter process is boosted by laparotomy. Evidently, lysis is concentrated in the area adjacent to the lesion, but may also take place at remote sites. It occurs particularly during the first postoperative week, after which, in the absence of sepsis or starvation, synthesis overcomes the lysis and the equilibrium is restored. Therefore, there is a risk of the aneurysm rupturing during this period. Subsequently, other authors have considered this to be speculative or doubtful. For some, its effect is minimal given that, in many situations, there is no direct trauma in the area of the aneurysm [10]. Others, however, cannot discount that the possibility that the risk factor may exist, although it does not affect all aneurysms equally, since it depends on the degree of aggression of the laparotomy and the size of the aneurysm [11].

Our patient presented the two aforementioned risk factors for rupture: (1) a large-diameter aneurysm and (2) stress on the wall (due to laparotomy and sepsis). Fortunately, the stress was not severe enough to trigger the rupture of the AAA during the postoperative period.

#### Preoperative risk prediction

Patterson *et al.* [12] performed a systematic review to examine and compare existing preoperative risk prediction methods for elective AAA repair. They reviewed 28 articles encompassing 10 risk models. The most frequent risk prediction models were the Glasgow Aneurysm Score (GAS), the Physiological and Operative Severity Score for enUmeration of Mortality (POSSUM) predictor equation, and the Vascular Biochemistry and Haematology Outcome Model (VBHOM). All models had strengths and weaknesses. According to this systematic review, the GAS appears to be the most useful and consistently validated score at present for open repair. Recent work has shown that no scores consistently predicted the risk associated with endovascular aneurysm repair (EVAR). Since 2015, we have used the AAA Score tool [13] in our department. This application, developed by Graeme Ambler on behalf of the British Society for Endovascular Therapy, implements preoperative risk prediction models, including endovascular procedures.

### Appendicitis in the elderly

Appendicitis is the most common reason for acute abdominal surgery in the general population. It is much more frequent in the young than in the elderly population, although it is becoming more common in the latter group because of their increasing life expectancy. The presentation of appendicitis (fever, pain in the right iliac fossa, etc.) in the elderly is not common, and for that reason the initial diagnosis is only correct in half of the cases [14]. While computed tomography may represent a useful diagnostic tool and laparoscopic appendectomy may be appropriate for selected patients, neither has affected outcomes when measured for morbidity and mortality rates [15]. The presence of atypical cases and the delay in diagnosis and surgery leads to an increase in the incidence of perforated appendicitis and higher mortality [16]. A recent series of 112 appendectomies in elderly patients showed a perforation and morbidity rate of 40% and 28%, respectively. There was no mortality [17]. Nevertheless, around half of appendicitis deaths occur in the elderly [1, 2].

In our patient, the clinical presentation of appendicitis was clearly atypical, and the CT, given the size and morphology of the AAA, led us to conclude symptomatic AAA with the risk of leakage or rupture, for which reason we recommended surgery. The possibility of a second pathology was not clearly suspected preoperatively. For all these reasons, our case is paradigmatic within appendicitis in the elderly: atypical clinical presentation, a CT scan of little value, and a perforated appendicitis.

### Association of AAA and appendicitis

AAA and various abdominal pathologies (cholelithiasis, colon cancer, appendicitis, etc.) are common in old age and, on rare occasions, may coexist. Although there are no figures in this regard, the finding of an AAA associated with another non-vascular intra-abdominal pathology area is increasingly commonly in the same patient. Under these circumstances, some surgeons will combine two operations in the same surgical session. Others limit themselves to treating the most serious pathology (usually, but not always, the AAA), leaving the other problem to be dealt with at a later time. This latter option could be considered the more prudent, since it is less complex and quicker, but it is also true that surgical aggression can exacerbate the untreated pathology. In any case, an optimal decision must seek to minimize: (1) the risk of rupture of the AAA after the first operation; (2) the risk of infection of the prosthesis, which is inserted when treating the aneurysm; (3) postoperative morbidity and mortality as a consequence of both pathologies [18]. As we will explain later, the introduction of endovascular surgery assuredly modifies these postulates.

Despite this, there is no consensus about the best option for achieving the stated objectives of minimising risk: Should everything be done the same operation or in sequential surgical acts; if the latter, which pathology should be treated first?

Although the therapeutic dilemma has not been resolved, the risks can be minimized when cases for simultaneous surgery are well chosen, and when staged surgery is decided upon, the first pathology to be treated and the time between the first and second stages are well chosen [19–22]. Sometimes the aggressiveness of the first operation makes it necessary to significantly delay the second surgery, or even prompts the patient to reject it [20, 21].

In general, the symptomatic lesion should be treated first. If both conditions are asymptomatic, the relative risks and benefits of treatment should be balanced against the likelihood that one or both conditions will become symptomatic [23]. However, there may be situations with two symptomatic pathologies, both of which may even require urgent treatment (e.g., complicated AAA plus perforated diverticula of the colon). Such combinations are particularly lethal and may require very aggressive synchronous actions.

Finally, we consider the endovascular aneurysm repair (EVAR) under these complex circumstances. This valuable tool could change the strategy by simplifying simultaneous treatment or by staging the procedures with a shorter delay [20].

For all the above reasons, we believe that perhaps the most important action is to individualize each case, as we have done with our patient. We initially intended to treat her AAA but changed our decision when we found perforated appendicitis associated with localized peritonitis. Unfortunately, our patient was not a candidate for EVAR.

In 1960, Ochsner, Cooley, and DeBakey [24] published the results of 480 "accidental" appendectomies in 931 patients with aneurysms without significant morbidity or mortality. We now know that "prophylactic" appendectomy provides little or no benefit and that it is not prudent to practice it in patients undergoing open surgery for AAA.

In 2000, Oshodi *et al.* [21], in their series of 55 patients with AAA coexisting with some other non-vascular abdominal pathology, reported two appendectomies for appendicitis, in which there was simultaneous treatment of AAA. However, the article does not provide any further useful information (for example, regarding the type of appendicitis or the possible appearance of complications in the postoperative period).

In 2009, Al Samaraee *et al.* [3] reported a case of AAA that was concomitant with acute appendicitis, an occurrence that, in the opinion of the authors, had never been published before. The patient, a 66-year-old man with acute abdominal symptoms, was diagnosed from a CT scan with acute appendicitis and infrarenal AAA (6.3 cm in diameter). Initially, open appendectomy (phlegmonous appendix) was performed; on a second occasion, after an interval of 10 days, and once the patient had fully recovered, open AAA surgery was performed. Thus, the symptomatic and life-threatening pathology was treated first, leaving, without discharging the patient, the treatment of the asymptomatic AAA but with a clear indication for surgery (diameter > 5.5 cm) for a later time. In the opinion of the authors, the simultaneous practice of both surgeries would have posed a risk of infection of the prosthesis.

Considering all the above, we are of the opinion that simultaneous surgery can be safe and cost-effective in cases of AAA and clean-contaminated (cholelithiasis) or even contaminated (with opening of the colon) surgery, but not in those of dirty surgery (abscess or peritonitis), as in the case reported here.

We think that our case is very different from that of Al Samaraee *et al.* [3], since the appendicitis changed from phlegmonous to perforated with local peritonitis. The case reported by Al Wahbi and Tamimi in 2015 [25] is similar to ours, where the patient, a 71-year-old man, had a huge AAA (10 cm diameter) concomitant with a diverticular abscess in the right iliac fossa. Initially, percutaneous drainage guided by ultrasound was performed in conjunction with antibiotic therapy (and other measures). Once this had resolved, on day 10, the authors carried out elective AAA open surgery. The patient was discharged after a further 10 days and showed no complications during a 3-year follow-up. The favorable incision-evacuation of the abscess and the successful medical treatment of diverticular disease were decisive in this case.

The relationship between AAA and appendicitis is rare and therefore scarcely described in the medical literature (Table 1). Its association modifies the clinical presentation (ruptured AAA that simulates appendicitis or the opposite as in our case) [26, 27] or induces serious complications, such as aneurysm [28–30] or endoprosthesis [31–34]. Finally, cases of primary [35] and secondary [36–38] aorto-appendicular fistulas have been described.

**Table 1:** Aortic abdominal aneurysm (AAA) and acute appendicitis (AAp)

Problem	No. of cases	References
AAA and AAp synchronic*	4	[3, 21]** and present case
Ruptured AAA simulating AAp	2	[26, 27]
AAp simulating a complicated AAA	1	Present case
Infection of an AAA by AAp	3	[28–30]
Infection of a Dacron prosthesis (AAA surgery) by AAp	4	[31]** [32] [33]
Infection of an EVAR prosthesis (AAA treatment) by AAp	1	[34]
Ruptured AAA induced by AAp	1	[30]
Primary aorto (AAA)-appendicular fistula	1	[35]
Secondary aorto (prothesis post-AAA surgery)-appendicular fistula	13	[36] [37]*** [38]

Literature review (*n* = 28 cases)

*EVAR* endovascular aneurysm repair

*Two options: (1) simultaneous surgery: risks (greater aggressivity; prosthesis infection) vs. benefits (no second surgery required); (2) surgery on two occasions (priority for the first time = risk of death from the pathology)

**Two cases

***Review (10 cases + 1 personal case)

## Conclusions

Abdominal aortic aneurysm and acute appendicitis are relatively common pathologies in elderly patients. However, the association of the two entities is very rare and serious. In general, the symptomatic lesion should be treated first. If both conditions are asymptomatic, the relative risks and benefits of treatment should be balanced against the likelihood that one or both conditions will become symptomatic. However, there may be situations with two symptomatic pathologies, both of which may even require urgent treatment. Such combinations are particularly lethal and may require very aggressive synchronous actions.

## Data Availability

The datasets used during the current study are available from the corresponding author on reasonable request.

## Abbreviations

CT: Computed tomography
AAA: Abdominal aortic aneurysm
MMPs: Matrix metalloproteinases
EVAR: Endovascular aneurysm repair
AAF: Aortoappendiceal fistula
